# Whole exome sequencing and system biology analysis support the "two-hit" mechanism in the onset of Ameloblastoma

**DOI:** 10.4317/medoral.24385

**Published:** 2021-01-04

**Authors:** Yueqi Shi, Mengyu Li, Yejia Yu, Yuqiong Zhou, Shaoyi Wang

**Affiliations:** 1 DDS, Department of Oral Surgery, Shanghai Ninth People's Hospital, College of Stomatology, Shanghai Jiao Tong University School of Medicine, National Clinical Research Center for Oral Diseases, Shanghai Key Laboratory of Stomatology and Shanghai Research Institute of Stomatology, China; 2 DDS, Professor, Department of Oral Surgery, Shanghai Ninth People's Hospital, College of Stomatology, Shanghai Jiao Tong University School of Medicine, National Clinical Research Center for Oral Diseases, Shanghai Key Laboratory of Stomatology and Shanghai Research Institute of Stomatology, China

## Abstract

**Background:**

Ameloblastoma is the most frequent odontogenic tumor. Various evidence has highlighted the role of somatic mutations, including recurrent mutation BRAF V600E, in the tumorigenesis of Ameloblastoma, but the intact genetic pathology remains unknown.

**Material and Methods:**

We sequenced the whole exome of both tumor tissue and healthy bone tissue from four mandibular ameloblastoma patients. The identified somatic mutations were integrated into Weighted Gene Co-expression Network Analysis on publicly available expression data of odontoblast, ameloblast, and Ameloblastoma.

**Results:**

We identified a total of 70 rare and severe somatic mutations. We found BRAF V600E on all four patients, supporting previous discovery. HSAP4 was also hit by two missense mutations on two different patients. By applying Weighted Gene Co-expression Network Analysis on expression data of odontoblast, ameloblast, and Ameloblastoma, we found a proliferation-associated gene module that was significantly disrupted in tumor tissues. Each patient carried at least two rare, severe somatic mutations affecting genes within this module, including HSPA4, GNAS, CLTC, NES, and KMT2D. All these mutations had a ratio of variant-support reads lower than BRAF V600E, indicating that they occurred later than BRAF V600E.

**Conclusions:**

We suggest that a severe somatic mutation on the gene network of cell proliferation other than BRAF V600E, namely second hit, may contribute to the tumorigenesis of Ameloblastoma.

** Key words:**Ameloblastoma, whole exome sequencing, somatic mutation, BRAF, HSPA4, two-hit theory.

## Introduction

Ameloblastoma is a benign, invasive odontogenic tumor with an average incidence rate of 0.92 per million person-years (1). Ameloblastoma consists of cells similar to ameloblast, which is responsible for depositing enamel during tooth development (2,3). Though rarely observed, Ameloblastoma has the potential of malignant transformation and become metastasizing Ameloblastoma and ameloblastic carcinoma with an incidence rate of 0.18 per million person-years (4). Even if the tumor does not transform, Ameloblastoma still exhibits local aggressiveness and the tendency to recurrence after surgical treatment (1), leading to a tremendous healthcare burden.

Currently, the mainstream management of Ameloblastoma is surgical resection. Due to the invasive potential and high recurrent rate, the surgical resection often has a margin of at least 1 cm from the margin of the tumor, leading to profound facial defects and morbidity (2). Conservative options like enucleation or curettage often result in a high recurrence rate (5,6). The alternative management of radiation therapy has a limited application in Ameloblastoma because of the risk of malignant transformation (2). To this end, the understanding of molecular mechanism of the disease is urgently desired so that etiological chemical treatment can be found.

The fundamental role of somatic mutations in the tumorigenesis of Ameloblastoma has been demonstrated by target sequencing (7) and transcriptomic analysis (3). One of the most significant discoveries is that a missense mutation on BRAF, V600E, recurrently occurs in most of the patients (8). BRAF encodes a serine-threonine kinase that conducts signal transduction of MAPK pathway. BRAF V600E mutation permanently activates this enzyme and subsequently downstream MEK and ERK signaling (7,9). Recently, Guan *et al*. (6) reported a Whole-Exome Sequencing (WES) study on Ameloblastoma and confirmed the influence of tobacco on the somatic mutation, supporting its role in the tumorigenesis.

So far, these genetic studies have identified mutations on several vital genes (BRAF, SMO, CTNNB1, etc.) and critical signaling pathways (MAPK pathway, Wnt pathway, etc.). An integrative approach to interpreting the biological significance of these mutations is necessary to explore the intact genetic etiology of Ameloblastoma. To achieve this goal, we performed WES on ameloblastoma patients and combined this genomic data with previously published (10,11) transcriptomic data to conduct a multi-omics system biology analysis. We confirmed that the biological significance of each isolated mutation could be interpreted from the aspect of gene network. With these efforts, we highlight the "two-hit" mechanism in the pathology of Ameloblastoma and suggest that all results from current genetic studies could be integrated using a systematic approach so that an entire picture of ameloblastoma genetics could be depicted.

## Material and Methods

- Patient Recruitment and Sample Collection

The current study was approved and supervised by the ethics committee of Shanghai Ninth Peoples Hospital affiliated to Shanghai Jiao Tong University, School of Medicine. It was carried out following The Code of Ethics of the World Medical Association (Declaration of Helsinki). Informed consent was obtained from all participants. We recruited four patients with Ameloblastoma on their mandible. We collected and fresh-frozen mandibular ameloblastoma tissues as well as normal bone tissues nearby from each patient during the operation. We extracted the total DNA from each sample by Pinpoint Slide DNA Isolation System (Zymo Research) according to the manufacturer's protocol.

- Whole Exome Sequencing

About 260ng of DNA from each sample was used to construct the pre-captured DNA library by DNA Seq Library Preparation Kit-Illumina Compatible (K02422, Gnomegen, San Diego, CA, USA) according to the manufacturer's instructions. The fragmented DNA was subsequently end-repaired, ligated to adaptors, and subjected to PCR amplification with 9 or 11 amplification cycles according to the manufacturer's protocol with several purification steps to get library products with different indexes (K02422, Gnomegen). The pre-captured library containing exome sequences was captured by SureSelect Capture Library kit (Agilent). The exome-enriched libraries were sequenced on the Illumina HiSeq 2000 platform with 1000× sequencing depth, and paired-end reads with an average size of 125 base pairs (PE125) were generated.

- Data Preprocessing

Sequencing data sequenced by HiseqTM Sequencer was filtered (removing the adaptor sequences, reads with >5% ambiguous bases (noted as N) and low-quality reads containing more than 20 percent of bases with qualities of <20) and mapped to Human genome Version GRCh37 Ensembl75 NCBI utilizing BWA-mem under following parameter (bwa mem -t 8 -R) (12). Duplicated reads were marker by PICARD, and recalibration was applied based on the GATK SNP standard calling pipeline tools.

- Variant Calling and annotation

SNV and Indel calling was achieved by Varscan v 2.3.6 (13). In brief, for each patient, we compared the number of reads that support each plausible SNV/Indel in tumor and normal tissue and calculated the *p-value* via the Fisher test. We annotated all variants by wAannovar (14) online tool. All synonymous mutations were discarded.

- Variant Filtration

**Figure 1:**
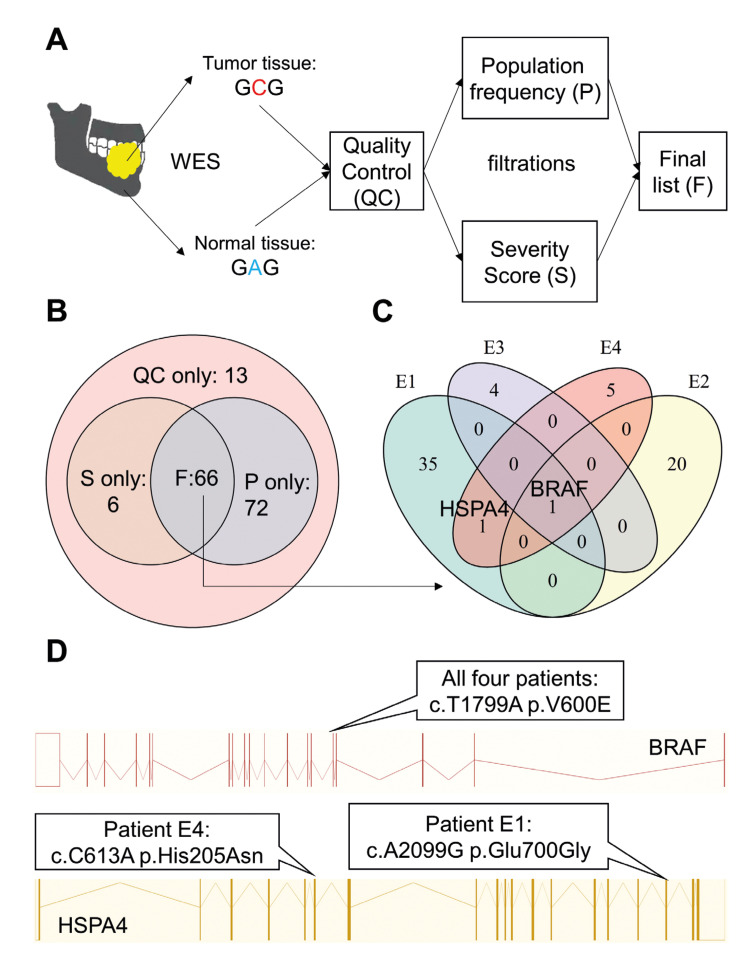
Somatic mutation profiles of Ameloblastoma. A: Schematic views of mutation filtration procedures. B: Venn diagram showing the number of genes hit by different types of somatic mutations as defined in A. C: Venn diagram showing the distribution of genes hit by final mutations among all four patients. D: Details of recurrent mutations. Upper panel: recurrent mutation on BRAF; lower panel: recurrent mutations on HSPA4.

We applied Quality Control (QC) by following criteria: *p-value* by Varscan<0,05; sequence depth ≥ 10 for both normal and tumor tissue; variant-supporting reads ≤ 1 for normal tissue and ≥ 3 for tumor tissue; frequency of variant-supporting reads ≤ 0.03 for normal tissue and ≥ 0.05 for tumor tissue. All variants that pass QC ("QC" in Fig. 1) were filtered by population frequency ("P" in Fig. 1) and predicted severity score ("S" in Fig. 1). We defined variants with population frequency < 0.005 in East Asia population from both 1000 Genome (15), ExAC (16) GnomAD (17) genome, and GnomAD exome database as rare mutations. Variants that were annotated as frameshift or stop site/start site/splice site mutations were considered severe. For the remaining missense variants, we considered those predicted to be damaging by at least two tools (CADD Phred score (18) ≥ 30, SIFT (19) prediction = D, Polyphen2 (20) prediction = D) were severe mutations. Mutations that were both rare ("P" in Fig. 1) and severe ("S" in Fig. 1) were included as the final mutation list ("F" in Fig. 1).

- Weighted Gene Co-expression Network Analysis (WGCNA)

We applied WGCNA (21) on a combined expression array dataset (GSE63829 (10) & GSE68531 (11)), which included data of normal odontoblast, ameloblast, and various types of Ameloblastoma. We used pickSoftThreshold function to choose a power of 12. Unsigned Topology Overlap Measure (TOM) was calculated based on the adjacency matrix, followed by dynamic hierarchical clustering with a cut height of 0.4. We calculated the first principle component of each co-expression modules, namely Module Eigengene (ME), to represent the expression level of each module. We calculated the correlation between ME and sample types (normal or tumor tissue) to find module (s) that significantly altered in tumor tissue. For each gene, we calculated the correlation between its expression and the ME of the module it belonged to. The correlation coefficient, kME, was used to find hub genes for each module.

- Network Analysis

For the turquoise module, we first found all genes that had a TOM ≥ 0.15 with a least one gene that carried a somatic mutation (F). A subnetwork of turquoise module was built by these genes, together with all genes that carried somatic mutation (F), as well as their connection (TOM ≥0.15). Network analysis was carried out in Cytoscape (22).

- Gene Ontology (GO) Analysis

We performed GO enrichment analysis of interested gene lists by ClusterProfiler R package (23). We tested if genes of interest enriched in any GO-BP pathway by hypergeometric test. Gene background was defined as all genes with GO annotation. Only pathways with ≥ten genes were included in our analysis. The P-value of hypergeometric tests was adjusted for multiple testing by the Benjamin-Hochberg method. For all pathways with adjusted *p-value* ≤0.05, we chose non-redundant pathways (no pathway was parent term of any other pathway) as our final results.

## Results

- Mutation profiles of Ameloblastoma patients showed significant heterogeneity

To control for confounders of onset age (6) and tumor position (3,6) that were previously found to affected mutation profiles of Ameloblastoma, we only included adult patients with Ameloblastoma on their mandible. We performed WES on these four patients and found a total of 1072 putative somatic mutations. After strict quality control and Varscan (13) mutation calling, we identified 175 somatic mutations that reached quality and significance threshold (87 on patient E1, 54 on patient E2, 14 on patient E3, 19 on patient E4), which hit 157 genes. We further filtered these mutations based on their population frequency and predicted severity by CADD (18), PolyPhen (20) and SIFT (19), which identified 151 rare mutations on 138 genes and 77 severe mutations on 72 genes. A total of 70 rare and severe mutations on 66 genes, referred to as "final mutation," were identified as the intersection of the above mutation lists (Fig. 1). The final mutation showed a significant unbalanced distribution among patients: patients E1 carried 37 rare, severe mutations, whereas patient E3 carried only five (Fig. 1). The coding consequence of mutations also varied among patients: only one nonsense mutation was found on E3 (disrupting GNAS) and E4 (disrupting TENM4), whereas E1 carried five nonsense mutations and five frameshift deletions. Gene Ontology (GO) analysis on Biological Pathway revealed that final mutations showed a tendency of enrichment in osteogenesis-related pathways (GO 0001649, osteoblast differentiation: *p*=0.005, p-adjust=0.32). This trend was less evident for rare mutations (GO 0001649: *p*=0.012) and severe mutations (GO 0001649: *p*=0.007). However, none of the enrichment reached the significance threshold (False Discovery Rate <0.05).

- Ameloblastoma patients carried recurrent somatic mutations on BRAF and HSPA4

Previous studies (3,24) have demonstrated that a missense somatic mutation on BRAF (V600E) frequently happened in Ameloblastoma. Furthermore, drugs that target this mutation showed the capacity to inhibit Ameloblastoma. In accordance with these findings, we found BRAF V600E in all four patients (Fig. 1). This missense mutation has never been found in the east Asia population. It is predicted to be highly damaging by all three algorithms (SIFT score: 0.001; Polyphen-2 score: 0.975; CADD-phred: 32), in agreement with the vital role of BRAF in EGFR signaling pathway (3,24). Another gene, HSPA4, was recurrently hit by missense somatic mutations (patient E1: E700G; patient E4: H205N. Fig. 1). One of the mutations, E700G, interrupted the antibody binding site that spanned from residual 699 to 808. Both mutations have never occurred in any population of 1000 Genome, ExAC or GnomAD. However, although they were both considered as damaging by our filtration, their predicted severity was not as high as BRAF V600E (E700G: Polyphen-2 score=0.455; H205N: CADD phred=29.6). By comparing our mutation list to a previous WES study by Guan *et al*. (6), we found that KMT2D was also recurrently affected by severe somatic mutations: we found a frameshift deletion on patient E1 that hit KMT2D, whereas Guan *et al*. found two nonsense mutations in two adult patients with mandibular Ameloblastoma.

- Gene co-expression network of cell proliferation pathways was disrupted in Ameloblastoma

**Figure 2:**
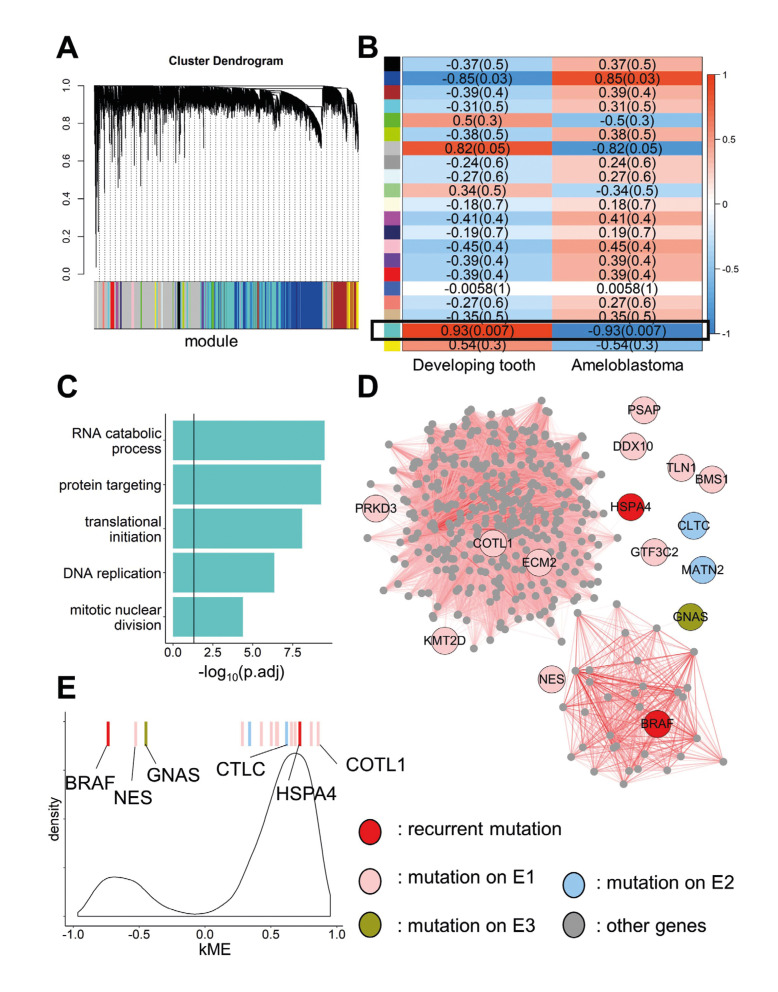
Network analysis of somatic mutations within ameloblastoma-associated co-expression module. A: Cluster dendrogram showing the partition of co-expression modules from WGCNA. B: Module-trait correlations between each module and sample type (normal tissue or ameloblastoma tissue). C: GO-BP enrichment results for genes within turquoise module. Vertical line indicates the significant threshold of p-adjust<0.05. D: Sub-network of turquoise module with all genes that connect to at least one mutation genes with TOM>0.15. All mutation genes are shown in large with gene symbols. Colors indicate the mutation origin. Transparency of edges correspond to Topology Overlap Measures. E: Distribution of module membership (kME) of all genes within turquoise module. Color bars indicate module membership of mutation genes.

So far, we have identified several key genes in Ameloblastoma. These genes, together with previous results of somatic mutations analysis (3,6), roughly depict the genetic etiology of Ameloblastoma. However, these results were yet separate findings without obvious convergence in terms of biological functions or pathways. We reasoned that an integration of the isolated findings by system biology approach would help to draw an intact picture. To achieve this goal, we first adopted transcriptome data of odontoblast, ameloblast, and Ameloblastoma from Hu *et al*. (10,11) and applied Weighted Gene Co-expression Network Analysis (WGCNA) on it. Hierarchical clustering analysis identified 21 co-expression modules on the combined transcriptome data (Fig. 2).

Using module-trait correlation analysis, we found that Module Eigengene (ME, first principle component of all genes within the module) for turquoise module was significantly associated with sample types (correlation coefficient = 0.93, *p*=0.007) (Fig. 2). Genes within turquoise module were profoundly down-regulated in tumor tissues without significant inter-tumor heterogeneity. GO-BP enrichment analysis revealed that turquoise module contained genes involved in proliferation-associated processes, such as RNA catabolic process (p-adjust=3.07×10-10), translational initiation (p-adjust=8.06×10-9) and mitotic nuclear division (p-adjust=4.18×10-5), etc.. Another module (blue) also exhibited a moderate correlation with sample type (correlation coefficient = 0.85, *p*=0.03) (Fig. 2). However, genes within blue module showed low expression levels and high variability. Furthermore, the only functional enrichment they had was the regulation of membrane potential (p-adjust=0.004). Considering the above results, we conclude that the gene network regulating cell proliferation exhibits profound dysregulation during tumorigenesis and should be recognized as the key component of Ameloblastoma pathology.

- Each Ameloblastoma patient carried at least two severe somatic mutations affecting the gene network of cell proliferation

Finally, we integrated the transcriptomic gene network with our genomic mutation profile to interpret the pathology of Ameloblastoma. A total of 16 rare, severe somatic mutations were found to affect genes within turquoise module (Table 1). Among them, we found genes with previously implicated recurrent mutations (BRAF, HSPA4, and KMT2D) and proto-oncogenes like NES and GNAS. For patient E1, 12 genes within turquoise module were hit by mutations. The remaining three patients also carried at least two mutations affecting this module.

Using Topology Overlap Measures (TOM) calculated by WGCNA, we built up a sub-network of turquoise module, which consisted of all genes with connection to mutation genes at TOM>0.15 (Fig. 2). A surprising fact was that this sub-network contained two separate small networks, one with 36 genes centered around BRAF (degree=31) and another with 366 genes around COTL1 (degree=304). We further calculated the correlation between each gene and ME, namely kME. We found that the first small network consisted of those genes negatively correlated with ME, and the second consisted of those positively correlated with ME (Fig. 2). As expected, BRAF (kME=-0.74) and COTL1 (kME=0.86) was the hub of each small network. Another two proto-oncogenes, GNAS (kME=-0.45) and NES (kME=0.53) were also negatively correlated with ME.

Another evident fact was that most of the mutation genes did not stand in the central position. Put BRAF aside, mutations from patient E2 hit genes with zero degree and low kME (CLTC: kME=0.62; MATN2: kME=0.38). Genes hit by mutation from E3 (GNAS) and E4 (HSPA4: kME=0.72) were also poorly connected (degree=1) (Fig. 2). Together with the previous observation that most Ameloblastoma occurred in adult (6) or mandible (3) carried BRAF V600E somatic mutation, we inferred that disruption of hub gene BRAF is essential, but not sufficient, to the tumorigenesis of adult mandibular Ameloblastoma. A second hit on genes within the network of cell proliferation, regardless of its position in the network, maybe the final trigger of the process of tumorigenesis. Supporting evidence was that in all four patients, the ratio of variant-support reads for BRAF V600E was higher than all other mutations affecting genes within turquoise module (Table 2), which indicated that BRAF V600E occurred earlier than other secondary mutations.

**Table 1:**
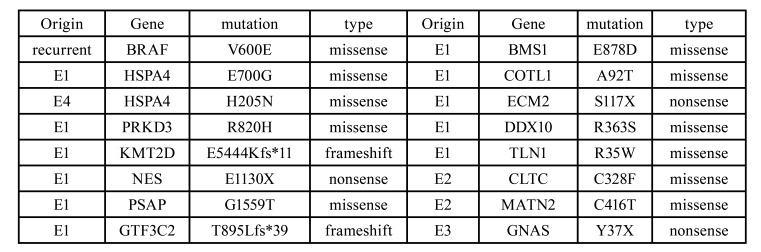
All somatic mutations that hit genes within turquoise module.

**Table 2:**
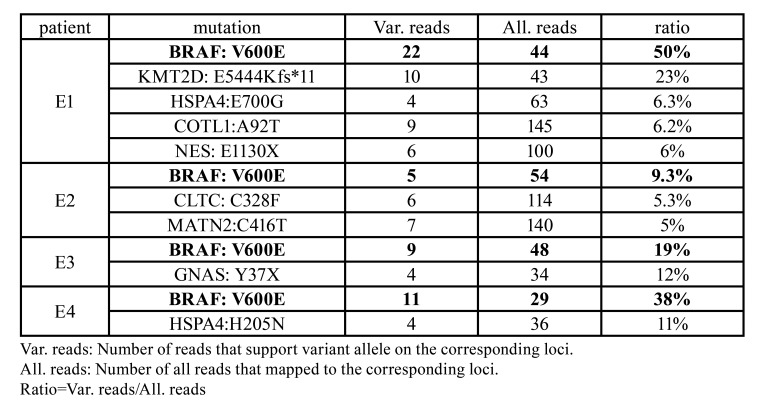
Ratio of variant-support reads for highlighted mutations in each patient.

## Discussion

In the current study, we reported an exome-wide somatic mutation profile for Ameloblastoma. We have shown that somatic mutations exhibited a profound heterogeneity between patients with little convergence on biological functions. Furthermore, only a few genes were recurrently affected by somatic mutations in different patients. Taken together, we concluded that somatic mutations on different patients were highly heterogeneous with little convergence and that the majority of these mutations did not directly contribute to the tumorigenesis. Thus, we suggest that the somatic mutations of Ameloblastoma should be studied from an integrative perspective so that an entire picture could be depicted.

One of the main findings of our study is the recurrent mutations on HSPA4. HSPA4, a member of Heat Shock Protein family (HSP), which act as molecular chaperones in conditions of stress and tumorigenesis, suppresses apoptosis and enhances the aggressiveness and prognosis of tumor tissue (25). The previous study has demonstrated its role in cancer like hepatocellular carcinoma (25), but no association between HSPA4 and Ameloblastoma has ever been reported. Our findings of recurrent somatic mutation on HSPA4 suggest that its proto oncogene-like function may play a role in the pathology of Ameloblastoma.

Taking one step further, we integrated all somatic mutations identified in the current study and suggested that the two-hit mechanism may play a role in the tumorigenesis of Ameloblastoma. First introduced by Dr. Knudson (26), the two-hit theory of carcinogenesis stated that one deleterious mutation on a proto-oncogene or tumor suppressor gene only caused the susceptibility to tumor, and a second mutation on relating gene was necessary to trigger tumorigenesis finally. This theory is in line with our observation of somatic mutations on Ameloblastoma from three aspects: first of all, there is a common mutation (BRAF V600E) shared by all four patients; secondly, all four patients carried other mutation (s) that also hit genes within the same gene network of BRAF, and this network regulates the vital process of cell proliferation; lastly, BRAF V600E occurred earlier than other mutations. In conclusion, we observed that all four Ameloblastoma patients carried BRAF V600E which maybe the first hit crucial for Ameloblastoma susceptibility, and they all carried a second mutation, even a less deleterious one, which may eventually start the tumorigenesis.
